# Assessment of Dysphonia in Children with Pompe Disease Using Auditory-Perceptual and Acoustic/Physiologic Methods

**DOI:** 10.3390/jcm10163617

**Published:** 2021-08-16

**Authors:** Kelly D. Crisp, Amy T. Neel, Sathya Amarasekara, Jill Marcus, Gretchen Nichting, Aditi Korlimarla, Priya S. Kishnani, Harrison N. Jones

**Affiliations:** 1 Department of Head and Neck Surgery & Communication Sciences, Duke University School of Medicine, Durham, NC 27710, USA; kelly.crisp@duke.edu; 2 Department of Speech and Hearing Sciences, University of New Mexico, Albuquerque, NM 87131, USA; atneel@unm.edu; 3 Duke Clinical Research Institute, Duke Health, Durham, NC 27710, USA; sathya.amarasekara@duke.edu; 4 Division of Speech Pathology and Audiology, Duke Health, Durham, NC 27710, USA; jill.marcus@duke.edu; 5 Department of Pediatrics, Division of Medical Genetics, Duke University School of Medicine, Durham, NC 27710, USA; gretchen.nichting@duke.edu (G.N.); aditi.korlimarla@duke.edu (A.K.); priya.kishnani@duke.edu (P.S.K.)

**Keywords:** pompe disease, speech, voice, dysphonia, acoustic, auditory-perceptual, GRBAS, respiratory

## Abstract

Bulbar and respiratory weakness occur commonly in children with Pompe disease and frequently lead to dysarthria. However, changes in vocal quality associated with this motor speech disorder are poorly described. The goal of this study was to characterize the vocal function of children with Pompe disease using auditory-perceptual and physiologic/acoustic methods. High-quality voice recordings were collected from 21 children with Pompe disease. The Grade, Roughness, Breathiness, Asthenia, and Strain (GRBAS) scale was used to assess voice quality and ratings were compared to physiologic/acoustic measurements collected during sustained phonation tasks, reading of a standard passage, and repetition of a short phrase at maximal volume. Based on ratings of grade, dysphonia was present in 90% of participants and was most commonly rated as mild or moderate in severity. Duration of sustained phonation tasks was reduced and shimmer was increased in comparison to published reference values for children without dysphonia. Specific measures of loudness were found to have statistically significant relationships with perceptual ratings of grade, breathiness, asthenia, and strain. Our data suggest that dysphonia is common in children with Pompe disease and primarily reflects impairments in respiratory and laryngeal function; however, the primary cause of dysphonia remains unclear. Future studies should seek to quantify the relative contribution of deficits in individual speech subsystems on voice quality and motor speech performance more broadly.

## 1. Introduction

Pompe disease, caused by a deficiency of the enzyme acid-alpha glucosidase (GAA), is characterized by an abnormal accumulation of glycogen in the lysosomes of multiple tissues, including skeletal, cardiac, and smooth muscles. Pompe disease is broadly classified into two groups: Infantile and late-onset Pompe disease. Infantile-onset Pompe disease (IOPD) represents the most severe end of the clinical spectrum. Children with IOPD present with hypertrophic cardiomyopathy and profound muscle weakness at or soon after birth. Symptom onset for patients with late-onset Pompe disease (LOPD) ranges from the first year of life to later adulthood. Individuals with LOPD generally exhibit a slower rate of disease progression and suffer less severe clinical outcomes than those with IOPD. Even within these categories, the disease exists along a continuum with variable clinical presentation related age of symptom onset, amount of residual GAA, and cross-reactive immune material (CRIM) status [1,2].

Since the introduction of enzyme replacement therapy (ERT) in 2006, children with IOPD are surviving longer [1]. The wide-spread adoption of newborn screening (NBS) programs has resulted in the identification of more children with Pompe disease, both with and without clinical symptoms. Accordingly, new phenotypes are emerging in the survivors of IOPD and children with LOPD. There is evidence of motor-based impairments that persist due to residual myopathy, including progressive skeletal muscle weakness, gait abnormalities, contractures, ptosis, and respiratory decline [3]. 

Dysarthria is a neuromuscular speech disorder in which damage to the central and/or peripheral nervous system or muscles affects speech production. Flaccid dysarthria results from weakness caused by damage to the motor unit and may arise from a variety of neurologic diseases and conditions including myopathy [4]. Characteristics of dysarthria associated with bulbar weakness include hypernasality, nasal emission, short phrases, reduced articulatory precision, and reduced speech intelligibility, all of which negatively impact affected individuals’ communication abilities [4]. Dysarthria arising from bulbar weakness often includes changes in voice such as breathiness, reduced loudness, and hoarseness, which further reduce communicative effectiveness [4]. Previous reports describe articulation disorders, hypernasality, and impaired speech intelligibility consistent with flaccid dysarthria in children with Pompe disease [5,6,7,8]. However, speech disorders have received less attention in the literature than other motor-based impairments. 

Though our clinical experiences suggest that dysphonia (abnormal vocal quality) is a common feature of dysarthria in children with Pompe disease, relatively little detailed information about the voice characteristics of this population is available. We previously identified the presence of dysphonia in 35% of auditory-perceptual assessments in 10 children with IOPD via retrospective analysis [5]. Szklanny and colleagues investigated laryngeal function and structure in ten adults and nine children with LOPD [9]. Based on electroglottography and acoustic analysis, vocal fold insufficiency attributed to laryngeal weakness was present in both groups, though these changes were greater in children than adults with LOPD.

Recent investigations have identified disease impact in both the central and peripheral nervous systems of individuals with IOPD and LOPD [10,11,12,13,14,15]. Understanding clinical signs resulting from neurological and motor impairments, both individually and in combination with each other, are critical to refining our understanding of disease phenotype. Speech disorders associated with neurological involvement result in activity limitations and participation restrictions that negatively impact quality of life for many children with Pompe disease and therefore merit investigation. Bulbar and respiratory weakness occur commonly in children with Pompe disease and frequently lead to dysarthria; however, associated changes in vocal quality are poorly described. In this study, our goal was to characterize the vocal function of children with Pompe disease using auditory-perceptual and physiologic/acoustic methods that permit objective quantification of various aspects of the acoustic signal. We expected that both auditory-perceptual and physiologic/acoustic assessments would reveal abnormalities in vocal function occur commonly in children with Pompe disease.

## 2. Materials and Methods

### 2.1. Participants

English-speaking participants between the ages of 5 and 18 years with a confirmed diagnosis of IOPD or LOPD were recruited from the Duke University Pompe Disease Clinic and Research Program as part of a larger study investigating cognitive and neurological pathologies in children with Pompe disease (Pro00072329). Exclusion criteria were inability to travel to Duke for study assessments or refusal of informed consent. Written consent for participation was given by the participants’ parents or legal guardians. Verbal assent was obtained from children 6 to 11 years of age, and additional written assent was obtained from children 12 years of age and older. The study was approved by the Duke University Institutional Review Board and conducted in accordance with the Declaration of Helsinki. 

### 2.2. Procedures

#### 2.2.1. Auditory-Perceptual Assessment

The GRBAS (Grade, Roughness, Breathiness, Asthenia, and Strain) scale is a widely used and highly reliable perceptual scale to assess voice quality in individuals with voice disorders [16,17]. Each of five voice characteristics is rated on a Likert scale of 0–3 in which 0 = normal/no disorder, 1 = mild disorder, 2 = moderate disorder, and 3 = severe disorder. Two speech-language pathologists (SLPs) with ten years or more of clinical experience listened to recordings of a vowel prolongation task and used the GRBAS scale to make judgments regarding voice characteristics. High-quality acoustic recordings of a series of speech tasks were obtained from each participant using a Sony PCM M-10 recorder and an omnidirectional Countryman head-mounted microphone. The microphone headframe and the mic boom were adjusted to achieve a consistent mouth-to-microphone position approximately 0.25″ to 0.5″ from the corner of the participant’s mouth when smiling. Analog signals were manually recorded at a sampling frequency of 44.1 kHz and 16 bit depth while the recording level and microphone sensitivity were held constant. The acoustic recordings were digitally encoded via linear PCM and saved on a microSD card, then transferred to a secure server on a lab computer where they were stored in WAV format. Lab personnel not otherwise involved in the research extracted recordings of the vowel prolongation task from the audio files of each study participant. Sample presentation was randomized using an online randomization sequence generator and the audio clips were compiled into a master audio file for auditory-perceptual assessment. In this master audio file, the vowel prolongation sample from each participant was presented five times in a row with a five-second break between each presentation, allowing raters to consider grade, roughness, breathiness, asthenia, and strain individually. A 10-s break followed the fifth presentation of each participant’s sample. 

Prior to collecting GRBAS scale ratings for analysis, the two raters completed a listener calibration session. After both raters reviewed the terms and definitions used in the GRBAS scale, approximately 20 samples of vowel prolongation were randomly selected from the data set. Each rater independently scored each sample using the GRBAS scale and then compared results, discussing the voice characteristics and their severity. The purposes of this training activity were to establish agreement regarding the definitions of the voice characteristics being evaluated and establish a joint reference for ratings of severity [18].

GRBAS scale ratings were collected for all participants in a single listening session. The master audio file was played in sound field for both raters simultaneously at a comfortable listening level over high-quality speakers in a quiet, carpeted room with <50 dB A of ambient noise. The raters scored the samples independently. After the listening session was completed, one of the two raters (HJ) compared the ratings for all samples and identified each GRBAS scale component that lacked exact agreement. One-month later, the two raters met again and re-listened to the samples in question. After discussing their impressions, a final consensus rating was recorded. 

#### 2.2.2. Physiologic/Acoustic Assessment

Instrumental assessment of voice was completed using the WEVOSYS lingWAVES measurement system and the lingWAVES Voice Protocol (version 3.2, WEVOSYS, Forchheim, Germany). Digital-acoustic voice data was collected using standardized hardware provided by the system manufacturer, which included a certified A meter/microphone set to C frequency and slow time weighting and the lingWAVES Connector USB containing its own high-quality sound card. The A meter/microphone was placed directly in front of the participant with the mic head 30 cm from the participant’s mouth. Data were collected in a quiet, carpeted room with ambient noise < 50 dB A. The lingWAVES Voice Protocol includes standard instructions for assessment tasks which included sustained phonation tasks, reading of a standard passage (the Rainbow Passage), and repetition of a short phrase at maximal volume. Participants completed all assessment tasks while seated. Participants who were unable to read aloud fluently were excluded from completing the oral reading task. A variety of measurements were derived, including /s/duration; /z/duration; s/z ratio; maximum phonation time (MPT); jitter; shimmer; mean fundamental frequency; mean loudness; glottal-to-noise excitation (GNE); and dysphonia severity index (DSI) for sustained phonation; mean, minimum, and maximum loudness for spoken text; and maximum loudness. Calculations for these parameters were automatically performed by the lingWAVES algorithm. There were no additional manipulations of the signal prior to calculation. 

### 2.3. Statistical Analysis

Descriptive statistics were calculated to describe the sample characteristics. Weighted kappa statistics were utilized to examine the interrater agreement of ordinal auditory perceptual ratings between the two raters. The results were assessed as <0 indicating less than chance agreement, 0.01–0.20 as slight agreement, 0.21–0.40 as fair agreement, 0.41–0.60 as moderate agreement, 0.61–0.80 as substantial agreement, and 0.81–0.99 as almost perfect agreement [19]. Multiple regression analysis models were used to assess the relationships among each voice protocol variable as the outcome and each auditory-perceptual feature as the predictor controlling for sex and age at assessment. The analyses were conducted using SAS/STAT software (version 9.4, SAS System for Windows, SAS Institute Inc., Cary, NC, USA, 2012). All analyses were two-tailed with a *p* < 0.05 deemed as statistically significant. 

## 3. Results

Auditory-perceptual and physiologic/acoustic voice data were collected from 21 children with Pompe disease with a mean age of 9.9 years (median = 9.4, SD = 3.7, range 5.0–17.0) at the time of assessment. Seventeen of 21 participants were diagnosed with IOPD; 14 were CRIM positive and three were CRIM negative. Four of 21 participants were diagnosed with LOPD. All participants were on ERT at the time of assessment; 4/21 received standard of care (20 mg/kg biweekly) and 16/21 received doses ranging from 30–40 mg/kg weekly/biweekly. Complete demographic data for participants with IOPD and LOPD are provided in Table 1. Additional cohort characteristics are contained within the Supplementary Materials (Table S1).

### 3.1. Inter-Rater Agreement

The weighted kappa for each coefficient is provided in Table 2. In the first listening session, moderate agreement was achieved between the two raters for grade (0.51, *p* < 0.01), breathiness (0.45, *p* = 0.02), asthenia (0.58, *p* < 0.01) and strain (0.57, *p* < 0.001). Fair agreement was achieved for roughness (0.36, *p* = 0.04) [20]. Overall, samples from 17 of 21 participants (81%) required the two raters to re-listen to the sample to achieve consensus for one or more GRBAS component scores. Across the 210 individual GRBAS component scores provided by the two raters in the first listening session, 172 (82%) were in exact agreement after the first listen whereas 38 (18%) required re-listening. Original, independent ratings differed by 1 scale value in 36 (95%) of disagreements and by 2 scale values in 2 (5%) of disagreements.

### 3.2. Auditory-Perceptual Ratings

Auditory-perceptual ratings of grade (a proxy for overall dysphonia severity), roughness, breathiness, asthenia, and strain for 21 participants are presented in Table 3. Based on ratings of grade, dysphonia was present during vowel prolongation in 19/21 participants with IOPD and LOPD (90%). Across all five components of the GRBAS, deviations from normal were most commonly rated as mild or moderate in severity. Deviations from normal were infrequently rated as severe in the IOPD group, and none of the five GRBAS components were rated as severe in the LOPD group.

### 3.3. Physiologic/Acoustic Data

Summary statistics for physiologic/acoustic voice data are presented in Table 4. Individual physiologic/acoustic data for each participant is provided in the Supplementary Material (Tables S2–S4). Some participants were unable to complete all assessment tasks due to difficulty following task instructions or limitations in literacy. Equipment malfunction interfered with collection of physiologic/acoustic data in one participant. 

Our data revealed mean sustained phonation time for the phonemes/s/, /z/, and /a/ was reduced in study participants when compared to published reference values [21,22,23,24,25,26,27,28,29,30]. Duration of sustained/s/ was shorter than the 8–12 s thresholds for typically developing children in 20/21 participants and sustained/a/duration was shorter than 8 s in 18/21 participants. Only one participant, however, had an s/z ratio greater than 1.45, the threshold value for typical children. The majority of participants (18/20) had elevated mean shimmer values compared to pediatric normative threshold of 5%, but only 7 of 20 had jitter values that exceeded 0.5% [20,28,29]. The mean GNE value was 0.5 compared to the value of 0.90 found in children without dysphonia) [31]. In our sample, GNE values were below this threshold in 18/20 subjects. The mean DSI value was −0.6 and these values ranged from 3.6 to −5.0. Pebbili and colleagues report mean DSI values in typically developing children without voice complaints to be 2.9 in males and 3.8 in females [32]. Based on these thresholds, DSI values were abnormal in 14/15 participants. Mean loudness values for the passage read aloud fell within the range of typical speakers reported by Corthals (69.39 dBA (4.08)) [22]. Seven of the 14 participants who completed the task had mean loudness values below 65 dB; two of those produced mean loudness values below 60 dB. Maximum loudness levels produced when participants repeated a short phrase as loudly as possible appeared consistent with pediatric norms reported by Weinrich et al. [23]. Three of the 21 participants produced maximum loudness values of less than 83 dB, below the range of typical children.

### 3.4. Relationship between Auditory-Perceptual and Acoustic Data

We examined the relationship among auditory-perceptual and physiologic/acoustic outcomes using multiple regression models while controlling for sex and age at time of assessment (Table 5 and Table 6). Data for participants with IOPD and LOPD were collapsed for statistical analysis as there were minimal differences in the physiologic/acoustic characteristics of the two groups. Statistically significant relationships were identified between loudness measures and auditory perceptual ratings of breathiness and asthenia, including: Mean loudness during spoken text and breathiness (*p* < 0.01) and asthenia (*p* < 0.05); minimum loudness during spoken text and breathiness (*p* < 0.01) and asthenia (*p* = 0.03); and maximum loudness during spoken text and breathiness (*p* = 0.03) and asthenia (*p* = 0.04). As loudness increased, breathiness and asthenia ratings decreased. Loudness during an isolated maximum performance task was significantly related to grade (*p* < 0.01), breathiness (*p* = 0.02) and asthenia (*p* = 0.01). In addition, the relationship between s/z ratio and strain was statistically significant (*p* = 0.02). As s/z ratio increased, strain ratings decreased. Relationships between consensus ratings and other instrumental data did not reach statistical significance.

## 4. Discussion

These data provide a detailed description of vocal function in children with IOPD and LOPD using both a validated auditory-perceptual rating scale (GRBAS) and physiologic/acoustic measures. With 21 unique participants, our report also describes the voice features of the largest cohort of children with IOPD and LOPD in the literature to date. 

Two experienced SLP raters achieved moderate-fair agreement in rating voice quality using the GRBAS scale, which is comparable to that reported in other studies [34]. Dysphonia was a common finding in children with IOPD and LOPD when using this scale to evaluate vocal quality during vowel prolongation. Across all 21 participants, dysphonia was present in 90% of the sample. Based on ratings of grade, one participant with IOPD and one participant with LOPD were not judged as dysphonic during vowel prolongation. Dysphonia was judged as mild or moderate in severity in more than 80% of participants with IOPD and mild in 75% of participants with LOPD. No voice quality feature was rated as severe in the LOPD group. Breathiness and roughness were the most prevalent voice quality features identified by the raters in participants with IOPD and LOPD. Asthenia was present in 4/4 participants with LOPD; however, strain was noted less frequently in comparison to participants with IOPD. 

Overall, our physiologic/acoustic data suggest that MPT, /s/duration, and /z/duration are reduced and shimmer is increased in children with both IOPD and LOPD when compared to published reference values for children without dysphonia. The most obvious differences between our sample of children with Pompe disease and reference values for typically developing children were noted in sustained phonation tasks. Mean MPT was 5.9 s (4.4), lower than the range of reference values reported in typically developing children [23,24,27,30,35]. According to Finnegan, MPT < 8 s in females and <9 s in males should be considered abnormal [27]. Mean duration for sustained phonation of /s/ and /z/phonemes (mean values of 2.9 s (2.9) and 3.7 s (3.1), respectively) was also reduced in comparison to published normative data [24,25,26]. Sustained phonation tasks, widely included in voice evaluations in both clinical and research settings, are intended to assess the integrity of the laryngeal and respiratory systems and the ability to coordinate respiration with phonation [24,36]. Airflow measures such as vital capacity have been linked to MPT [28,37] and recent publications have recommended the inclusion of pulmonary function tests in voice assessment [38]. Respiratory muscle weakness with early involvement of the diaphragm is a known complication of both IOPD and LOPD [1,39,40] and therefore our finding of reduced MPT in this sample of children with Pompe disease is not surprising.

The integrity of laryngeal valving, neuromuscular control of the larynx, and its ability to rapidly adjust to various configurations of the vocal tract are also related to performance on sustained phonation tasks [28,36,37]. The s/z ratio task compares the duration of sustained production of /s/, a consonant that does not require vocal fold vibration, to the duration of sustained production of /z/, a consonant that does require vocal fold vibration. Typical speakers are expected to produce ratios below 1.4, sustaining both consonants for roughly the same amount of time, while speakers with vocal fold pathology may have ratios above 1.4 due to increased ability to sustain the voiceless/s/ compared to the voiced/z/. In this study, while the duration of /s/ and /z/ were reduced in our sample, the s/z ratio, an indicator of glottal efficiency, was below the 1.4 threshold for all but one participant. This may indicate that respiratory function had a larger impact than laryngeal function on total duration of sustained phonation in these participants. Interestingly, eight of the participants produced s/z ratios of less than 0.6; that is, they sustained the voiced/z/ for much longer than the voiceless/s/. This pattern may reflect a complex interaction between laryngeal function, voluntary control of the articulators influencing the shape and size of the vocal tract, and respiratory support for voicing [36].

Vocal intensity, or loudness, is also known to be impacted by pulmonary function, airflow measures, and neuromuscular control of the larynx [37,38,41]. Reference values for loudness are limited by variability in the way in which intensity is measured. The literature generally reports the intensity of conversational speech to vary between 50 and 70 dBA [42]. Corthals measured mean sound pressure over time (Leq) while 92 children between 7 and 18 years of age read the Rainbow Passage [22]. The participants in our study read the Rainbow Passage aloud with a mean loudness of 65.3 dBA (4.7), reflecting function at the lower end of the range reported by Corthals (65.31 to 73.47 dBA). However, performance varied substantially across individual participants, with loudness values during spoken text ranging from 56.7 to 73.5 dBA. It is the authors’ clinical impression that both overall loudness and loudness range are frequently reduced in children with Pompe disease.

Jitter and shimmer are objective acoustic measures of voice quality, indicating irregularities in vocal fundamental frequency and intensity. While jitter values for the participants in this study were within normal limits, mean values for shimmer, reflecting cycle-to-cycle variability in amplitude, were increased in our sample compared to published norms [29,30]. While increased shimmer may reflect vocal pathology, recent studies have shown that both shimmer and jitter are influenced by vocal loudness [43]. Less intense voices, like those of children with Pompe disease, are associated with higher shimmer and jitter values than louder ones.

Auditory-perceptual ratings of breathiness, asthenia, and grade were negatively correlated with loudness during spoken text and maximum loudness during an isolated maximal performance task. In other words, as loudness and glottal closure increased, perception of breathiness and asthenia decreased and grade, a proxy for overall dysphonia severity, improved. This suggests that participants with louder voices and more complete glottal closure were perceived to have less severe dysphonia; breathy and/or asthenic voices are unlikely to be loud. As noted above, mean loudness in our sample was comparable to available reference values for loudness in typically developing children [22,23]. Several types of acoustic measures were obtained, including measures of irregularity of vocal fold vibration (jitter, shimmer), inharmonic noise (GNE) and composite measures (DSI), but none were significantly related to auditory-perceptual ratings for these participants.

Prior descriptions of the speech and swallowing function of children with IOPD confirm that dysarthria and dysphagia are common and appear related to widespread involvement of the bulbar muscles [5,6,7,8,44,45,46,47]. Involvement of the central and/or peripheral nervous systems can influence bulbar muscle pathology and impact speech production [4,11,12,13,14,15,16]. The resulting signs and symptoms manifested in respiration, phonation, articulation, resonance, and prosody result in dysarthria that frequently persists despite speech treatment [8]. Hearing loss is also documented and may further impact speech [48] but does not fully explain the degree of speech impairment observed in affected patients [46]. Early diagnosis with early initiation of ERT [49], high-dose regimens of ERT [3,50], and adjunctive treatments like physical therapy and beta-2 adrenergic agonists [51] often result in improvements or stabilization of motor and pulmonary function. However, dysarthria frequently appears to remain. This study focused on vocal function in children with Pompe disease and our findings suggest that dysphonia primarily reflect impairments in respiratory support and laryngeal function. However, its clinical presentation is complex and the primary cause of dysphonia remains unclear.

Reduced duration of sustained phonation tasks suggests respiratory support compromised task performance, both in our study as well as in a detailed report of the speech and oromotor features of a cohort of 14 children with Pompe disease by Su and colleagues [7]. However, mean MPT of the children in our sample (5.9 s (4.4)) was shorter in duration than the mean MPT reported by Su (8.29 s (3.7)). The 12 children with IOPD in Su’s cohort were all identified by NBS with ERT initiation within one month of birth; CRIM status of these children was not reported. In the 17 children with IOPD from our sample, 11 were diagnosed >1 month of age, the median age at start of ERT was three months (range 0–13 months), and three of the participants were CRIM negative. As noted above, early initiation of ERT has been reported to have a positive impact on pulmonary function [3,49,52], which would be expected to improve respiratory support for phonation. Furthermore, the children with IOPD in our sample (mean 8.9 years (3.8), range 5.0–17.0) were older than the children with IOPD in Su’s cohort (mean 5.9 years (1.8), range 3.5–8.8). Since both respiratory muscle strength and sustained phonation duration are known to increase with age [53,54,55], this may indicate the children in our cohort had greater respiratory muscle weakness than those studied by Su.

The relationship between acoustic and auditory-perceptual analyses of voice quality for children with IOPD has not previously been explored; however, data describing the voice characteristics of children with LOPD are available for comparison and also provide evidence of laryngeal involvement. Szklanny and colleagues collected perceptual ratings using the GRBAS scale along with video-laryngoscopic examination, electroglottography, and acoustic recordings from 9 individuals with LOPD ranging from 7.5 to 25.6 years old [9,56]. Evidence of tense voice type, altered pitch, and dysphonia related to glottal insufficiency with incomplete focal fold closure during phonation was identified through video-laryngoscopic examination. However, overall grade was judged as normal in 75% (6/8) of ratings; mild or moderate breathiness, asthenia or strain were identified in 63% (5/8). In contrast, GRBAS scores from our cohort indicate both a higher rate of occurrence and greater severity of dysphonia. Overall grade was rated as normal in only 2/21 (9.5%) of our participants, while breathiness was present in >95%, roughness in >90%, strain in >70%, and asthenia in >65%. Diagnosis could account for this discrepancy, as 17/21 children in our sample were diagnosed with IOPD and could therefore be expected to present with greater disease severity than children with LOPD.

While clinicians might expect disease phenotype to have some relationship to the presence and severity of dysphonia, the small sample size of our study overall (*n* = 21) as well as the unequal distribution of participants with IOPD (*n* = 17) and LOPD (*n* = 4) precluded statistical analysis of such a relationship. Some of our acoustic and auditory-perceptual data suggest the presence of a relationship between disease phenotype and dysphonia severity and merit further study. For example, duration of sustained phonation tasks, DSI, and severity of overall grade ratings suggest the presence of more significant dysphonia in our participants with IOPD than those with LOPD. Longitudinal assessment of speech and voice characteristics within and across a larger sample of patients over time is needed to better understand the developing phenotypes of IOPD and LOPD.

Our findings suggest that the GRBAS scale can be used clinically to identify dysphonia in children with Pompe disease. We elected to use the GRBAS scale for auditory-perpetual assessment due its reliability and validity, widespread use in both clinical and research settings, and ease of administration. However, other scales such as the CAPE-V should be considered in future research. Compared to the GRBAS, the CAPE-V may be a better tool for the auditory-perceptual assessment of voice quality due to slightly improved intra- and inter-rater reliability, ability for its use in parametric statistical analysis, and the incorporation of additional parameters (e.g., pitch, loudness) which may enhance understanding of voice patterns [17].

The findings also emphasize the importance of collecting both auditory perceptual and physiologic/acoustic data when assessing voice as these measures provide complementary information about the presence and severity of dysphonia that will guide development of a treatment plan. The relationship between loudness during spoken text and maximum loudness during an isolated maximal performance task was statistically significant for dysphonia severity (overall grade) as well as ratings of breathiness and asthenia. It is possible that efforts to improve respiratory support, such as respiratory muscle training, combined with behavioral techniques to increase breath support and loudness during speech production may reduce the perceived severity of dysphonia in some children with Pompe disease. Loudness is an acoustic variable that is quick and easy to measure in most clinical settings and may be a useful objective data point to track alongside changes in perceptual ratings.

Alternative explanations for our findings and limitations of the present study must be considered. One limitation of this study was the relatively limited range of dysphonia severity present in our subjects, as 12 of 21 were judged to have normal voice quality or mild dysphonia. However, the range of dysphonia severity in this sample was greater than in previous research in this area [9,56]. Though our data reflect moderate-fair inter-rater agreement on GRBAS ratings, we did not assess intra-rater reliability. 

It is possible that we failed to capture accurate physiologic/acoustic data and identify relationships between auditory-perceptual and acoustic parameters due to measurement error, reduced participant effort, or the use of relatively novel equipment lacking robust age- and gender-specific norms. We attempted to interpret our acoustic/physiologic data using reference values reported by other investigators; however, thresholds for acoustic parameters differ among studies based on the analysis methods and algorithms employed by the equipment used for data collection [57]. This may limit the validity of our comparisons between the acoustic parameters collected from our participants and threshold values reported by other authors for typically developing children. Furthermore, sex, age, and puberty stage as well as differences in recording environment, assessment tasks, and task instructions are known to impact acoustic findings and therefore limit comparison of findings among studies [23,36,57,58,59,60]. For example, both shimmer and jitter have been shown to be influenced by vocal loudness; analysis of quieter voices may artificially inflate jitter and shimmer values [43].

Barties and De Bodt point out that a major limitation of many studies is the lack of correspondence between acoustic data collected from sustained phonation tasks and acoustic data collected during running speech [57]. Recent recommendations for preferred practice patterns for instrumental assessment from the American Speech-Language-Hearing Association endorse the use of connected speech tasks for analysis of habitual loudness, fundamental frequency range, and noise in the acoustic signal [61]. Cepstral-based measures, such as cepstral peak prominence (CPP), long-term averaged spectral measurements such as low-versus high-spectral ratio (LHR), and the cepstral and spectral index of dysphonia (CSID) may be better correlated with auditory-perceptual judgments of dysphonia than time-based spectral measures such as jitter and shimmer [62,63,64]. These analyses will be used in subsequent studies. Inclusion of laryngeal videostroboscopy and aerodynamic measures are also recommended for comprehensive instrumental assessment of dysphonia [61,65,66]; however, these measures were not collected in this preliminary study. We did not assess puberty stage in our male participants, which is known to affect fundamental frequency [67]. Though all audio recordings were obtained using consistent techniques in the same environment, recording in a sound booth or with a head-mounted microphone would have strengthened the quality of our data by optimizing the signal-to-noise ratio [57]. While correlations between physiologic/acoustic data and perceptual voice features have been identified by some authors [31,68,69], vocal quality is a multidimensional perceived construct and evidence of these correlations in both adults and children is inconsistent [57,70]. These and other data support the idea that neither auditory-perceptual nor physiologic/acoustic measures can stand alone, and a battery approach to clinical assessment of voice is necessary to fully describe the features of dysphonia, the extent of its functional impact, and evidence of benefit from intervention [70,71,72].

While these findings extend our knowledge of the voice characteristics of children with Pompe disease, we were unable to associate the presence and severity of dysphonia with impairment in a particular speech subsystem. Lack of respiratory and nasalance data limited our ability to attempt such an analysis. Future research should seek to quantify the relative contributions of deficits in resonance, respiration, and phonation to overall dysphonia severity. For example, useful insights may be obtained by comparing relationships among measures of pulmonary function and the acoustic and instrumental parameters that reflect the contribution of the respiratory system, such as MPT and loudness. Similarly, videostroboscopy or electroglottography should be utilized to provide additional information about the pattern of vocal fold vibration and glottal closure that could be associated with acoustic findings. Additional acoustic parameters such as the normalized amplitude quotient (NAQ), peak slope (PS), cepstral peak prominence (CPP), and harmonic richness factor (HRF) have shown value in prior research investigating the effects of Pompe disease on voice function and should be included in future research to better differentiate and describe dysphonic voices [9,56]. Hypernasality is widely reported to be the most commonly occurring deviant speech feature in children with Pompe disease [5,7,8,73] and the relationship between disorders of resonance and reduced speech intelligibility in other populations is well documented [30,74]. Quantifying the relative impact of deficits in individual speech subsystems in children with Pompe disease who exhibit dysarthria and dysphonia might allow clinicians to focus their interventions to maximize benefit from therapy and achieve optimal clinical outcomes. This is an important goal, as the presence of a communication disorder negatively impacts quality of life for many children with Pompe disease. Use of a patient-reported outcome tool such as the VHI-10 may provide additional insight into the functional impact of dysphonia on communication and should be included in future studies.

## 5. Conclusions

In summary, this study reveals that dysphonia is common in children with Pompe disease, and symptoms appear primarily related to dysfunction in the respiratory and laryngeal systems. However, with the exception of specific measures of loudness, the predictive relationship between our physiologic/acoustic data and auditory perceptual ratings was poor. The impact of dysfunction spread across the motor speech system is nearly certain and likely confounded our efforts to determine associations between auditory perceptual and acoustic voice data. The complex interrelationship between the various subsystems supporting voice production should be evaluated by adding electroglottographic, nasalance, and respiratory assessments. Comparison of these findings to measures of articulation and speech intelligibility will paint a more complete picture of speech disturbances in children with Pompe disease.

## Figures and Tables

**Table 1:** Baseline characteristics. Categorical variables presented as *n*, (%). Continuous variables presented as Mean (SD), median (Min-Max).

	IOPD (*n* = 17)	LOPD (*n* = 4)
**Sex**		
Male	8/17 (47%)	3/4 (75%)
Female	9/17 (53%)	1/4 (25%)
**Race**		
Caucasian	11/17 (65%)	2/4 (50%)
Black or African American	4/17 (24%)	1/4 (25%)
Asian	1/17 (6%)	1/4 (25%)
Other or more than one race	1/17 (6%)	-
**Ethnicity**		
Not Hispanic or Latino	15/17 (88%)	4/4 (100%)
Hispanic or Latino	2/17 (12%)	-
**Age at diagnosis (years)**	0.3 (0.3), 0.2 (0.0–1.1)	5.0 (4.5), 3.9 (1.1–11.1)
**Age at assessment (years)**	8.9 (3.8), 7.0 (5.0–17.0)	11.8 (2.2), 12.0 (9.0–14.0)
**CRIM status**		
Positive	14/17 (82%)	-
Negative	3/17 (18%)	-
**ERT history**		
ERT Start Age (years)	0.3 (0.3), 0.3 (0.0–1.1)	5.3 (4.7), 4.0 (1.4, 11.7)
Time on ERT at assessment (years)	9.0 (3.8), 7.6 (5.0–16.9)	7.1 (5.1), 7.9 (0.1–12.3)
**ERT dose at assessment**		
20 mg/kg biweekly	2/17 (12%)	2/4 (50%)
30 mg/kg weekly	1/17 (6%)	-
40 mg/kg biweekly	4/17 (24%)	-
40 mg/kg weekly	9/17 (53%)	2/4 (50%)
Infused biweekly; dose not recorded	1/17 (6%)	-

IOPD = infantile-onset Pompe disease; LOPD = late-onset Pompe disease; CRIM = cross-reactive immunological status; ERT = enzyme replacement therapy.

**Table 2:** Interrater agreement for GRBAS scale. Kappa interrater agreement between two listeners in the first listening session across 21 participants.

	Weighted Kappa	95% CI	*p*-Value	Disagreements by 1 Scale Value	Disagreements by 2 Scale Values
Grade	0.51	(0.23, 0.79)	0.00 *	7	1
Roughness	0.36	(0.07, 0.64)	0.04 *	8	0
Breathiness	0.45	(0.13, 0.77)	0.02 *	8	0
Asthenia	0.58	(0.32, 0.85)	0.00 **	8	0
Strain	0.57	(0.26, 0.87)	0.00 ***	5	1

* *p* < 0.05, ** *p* < 0.01, *** *p* < 0.001.

**Table 3:** Auditory-perceptual ratings of vocal quality.

	Normal	Mild	Moderate	Severe	Mean (SD)
IOPD (*n =* 17)					
Grade	1/17 (6%)	7/17 (41%)	7/17 (41%)	2/17 (12%)	1.59 (0.80)
Roughness	2/17 (12%)	9/17 (53%)	5/17 (29%)	1/17 (6%)	1.29 (0.77)
Breathiness	1/17 (6%)	11/17 (65%)	4/17 (24%)	1/17 (6%)	1.29 (0.69)
Asthenia	7/17 (41%)	6/17 (35%)	3/17 (18%)	1/17 (6%)	0.88 (0.93)
Strain	3/17 (18%)	13/17 (76%)	1/17 (6%)	-	0.88 (0.49)
LOPD (*n =* 4)					
Grade	1/4 (25%)	3/4 (75%)	-	-	0.75 (0.50)
Roughness	-	4/4 (100%)	-	-	1.00 (0.00)
Breathiness	-	3/4 (75%)	1/4 (25%)	-	1.25 (0.50)
Asthenia	-	4/4 (100%)	-	-	1.00 (0.00)
Strain	3/4 (75%)	1/4 (25%)	-	-	0.25 (0.50)
Overall (*n =* 21)					
Grade	2/21 (10%)	10/21 (48%)	7/21 (33%)	2/21 (10%)	1.43 (0.81)
Roughness	2/21 (10%)	13/21 (62%)	5/21 (24%)	1/21 (5%)	1.24 (0.70)
Breathiness	1/21 (5%)	14/21 (67%)	5/21 (24%)	1/21 (5%)	1.29 (0.64)
Asthenia	7/21 (33%)	10/21 (48%)	3/21 (14%)	1/21 (5%)	0.90 (0.83)
Strain	6/21 (29%)	14/21 (67%)	1/21 (5%)	-	0.76 (0.54)

Data presented as *n*, (%). Mean (SD) calculated where 0 = normal, 1 = mild, 2 = moderate, 3 = severe. IOPD = infantile-onset Pompe disease; LOPD = late-onset Pompe disease.

**Table 4:** Summary statistics for physiologic/acoustic data.

	Task	All Participants (*n* = 14 ^†^, 15 ^x^, 20 ^‡^, or 21)	Group-Wise Analysis	
			IOPD (*n* = 10 *, 12 **, 16 ^§^, or 17)	LOPD (*n* = 3 ^††^, 4)
Sustained phonation tasks [21,22,23,24,25,26,27,28,29,30,31,32,33]	/s/duration (s)	2.9 (2.9); 0.5–9.4	2.6 (2.8); 0.5–9.4	4.0 (3.1); 1.4–8.3
	/z/duration (s)	3.7 (3.1); 0.7–11.3	3.5 (3.1); 0.7–11.3	4.5 (3.7); 1.0–9.5
	s/z ratio	0.8 (0.4); 0.3–2.0	0.7 (0.3); 0.3–1.1	1.1 (0.7); 0.5–2.0
	MPT (s)	5.9 (4.4); 0.7–14.6 ^‡^	5.5 (4.5); 0.7–14.6	8.0 (3.6); 5.8–12.1 ^††^
	Jitter (%)	0.7 (0.8); 0.1–2.8 ^‡^	0.8 (0.9); 0.1–2.8 ^§^	0.2 (0.1); 0.1–0.3
	Shimmer (%)	11.2 (8.9); 4.9–40.6 ^‡^	11.9 (9.8); 5.0–40.6 ^§^	8.3 (2.5); 4.9–10.4
	Mean F0 (Hz)	244.6 (67.7); 114.5–351.2 ^‡^	246.5 (72.8); 114.5–351.2 ^§^	237.1 (49.2); 202.9–309.6
	Mean loudness (dBA)	76.8 (7.8); 63.0–93.1 ^‡^	77.7 (6.9); 66.5–93.1 ^§^	73.3 (11.3); 63.0–89.1
	GNE	0.5 (0.4); 0.2–2.2 ^‡^	0.5 (0.5); 0.2–2.2 ^§^	0.5 (0.3), −0.1–0.8
	DSI	−0.6 (2.2); −5.0–3.6 ^x^	−0.9 (2.4); −5.0–3.6 **	0.3 (0.5), −0.1–0.8 ^††^
Spoken text (Rainbow Passage) [22]	Mean loudness (dBA)	65.3 (4.7); 56.7–73.5 ^†^	65.9 (4.9); 56.7–73.5 *	63.9 (4.4); 59.2–68.8
	Max loudness (dBA)	71.9 (5.1); 65.0–81.2 ^†^	72.8 (4.9); 65.3–81.2 *	69.7 (5.6); 65.0–77.0)
	Min loudness (dBA)	55.7 (5.2); 46.0–61.8 ^†^	55.8 (5.3); 46.0–61.8 *	55.4 (5.6); 47.5–59.6
Max loudness task [23]	Max loudness (dBA)	91.3 (10.8); 58.9–104.3	89.9 (11.1); 58.9–103.9	96.9 (7.8); 89.6–104.3

Data are presented as mean (SD); range. MPT = maximum phonation time, s = seconds, Hz = Hertz, min = minimum, max = maximum, dBA = decibels A-weighted (reference value = 20 µPa), Fo = fundamental frequency; GNE = glottal-to-noise excitation; DSI = dysphonia severity index. Note mean values that differ from published normative data for typically developing children are highlighted in gray. ^†^ *n* = 14, ^x^ *n* = 15, ^‡^ *n* = 20; * *n* = 10, ** *n* = 12, ^§^ *n* = 16; ^††^ *n* = 3.

**Table 5:** Relationship between physiologic/acoustic voice data from sustained phonation tasks and consensus ratings from GRBAS scale, controlled for age at assessment and sex.

Versus GRBAS		Sustained Phonation Tasks									
		/s/ Duration (s)	/z/ Duration (s)	s/z Ratio	MPT (s)	Jitter (%)	Shimmer (%)	Mean F0 (Hz)	Mean Loudness (dBA)	GNE	DSI
G	Mean Est	0.76	0.63	0.01	−0.30	0.44	3.93	−0.94	−3.26	0.09	−0.18
	*p*-value	0.30	0.45	0.89	0.83	0.07	0.13	0.96	0.21	0.56	0.70
R	Mean Est	0.68	0.90	−0.07	1.12	0.20	0.15	−11.16	−1.90	−0.01	−0.34
	*p*-value	0.37	0.29	0.46	0.43	0.46	0.96	0.55	0.50	0.97	0.48
B	Mean Est	−0.47	−0.88	0.03	−2.20	0.21	−2.08	27.82	−5.84	0.26	−0.82
	*p*-value	0.63	0.42	0.83	0.21	0.56	0.59	0.27	0.12	0.25	0.16
A	Mean Est	0.05	−0.30	0.09	−0.89	0.00	−3.35	10.39	−4.19	0.21	−0.15
	*p*-value	0.95	0.70	0.30	0.49	0.99	0.20	0.57	0.11	0.18	0.73
S	Mean Est	0.20	1.02	−3.0	3.19	0.57	−0.42	−26.28	0.27	−0.07	−0.24
	*p*-value	0.85	0.39	0.02 *	0.11	0.09	0.91	0.28	0.94	0.75	0.73

G = grade, R = roughness, B = breathiness, A = asthenia, S = strain; Mean Est = mean estimate; s = seconds; MPT = maximum phonation time; SV = sustained vowel; F0 = fundamental frequency; Hz = Hertz, dB A = decibels sound pressure level A-weighted (reference value = 20 µPa); GNE = glottal-to-noise excitation; DSI = dysphonia severity index. * *p* < 0.05. Note mean values that differ from published normative data for typically developing children are highlighted in gray.

**Table 6:** Relationship between acoustic voice data from spoken text and maximal loudness tasks and consensus ratings from GRBAS scale, controlled for age at assessment and sex.

Versus GRBAS		Spoken Text (Rainbow Passage)			Maximum Loudness Task
		Mean Loudness (dBA)	Min Loudness (dBA)	Max Loudness (dBA)	Max Loudness (dBA)
G	Mean Est	−1.65	−2.65	−0.82	−9.39
	*p*-value	0.41	0.25	0.72	0.00 **
R	Mean Est	−0.67	0.27	−0.67	1.31
	*p*-value	0.73	0.91	0.76	0.71
B	Mean Est	−6.55	−7.68	−6.26	−9.57
	*p*-value	0.00 **	0.00 **	0.026 *	0.02 *
A	Mean Est	−4.59	−4.68	−4.44	−7.23
	*p*-value	0.01 *	0.03 *	0.04 *	0.01 *
S	Mean Est	4.69	5.01	4.63	6.5
	*p*-value	0.13	0.17	0.20	0.17

G = grade, R = roughness, B = breathiness, A = asthenia, S = strain; Mean Est = mean estimate; dB A = decibels sound pressure level A-weighted (reference value = 20 µPa); Min = minimum; Max = maximum. * *p* < 0.05, ** *p* < 0.01. Note mean values that differ from published normative data for typically developing children are highlighted in gray.

## Data Availability

The data presented in this study are available in the Supplementary Materials.

## Supplementary Materials

The following are available online at https://www.mdpi.com/article/10.3390/jcm10163617/s1, Table S1: Additional cohort characteristics; Table S2: Physiologic data from sustained phonation tasks for individual study participants; Table S3: Acoustic data from sustained phonation tasks for individual study participants; Table S4: Acoustic data from spoken text and maximal loudness tasks for individual study participants.
