# Supplementary material for: Assessment of Dysphonia in Children with Pompe Disease Using Auditory-Perceptual and Acoustic/Physiologic Methods
Source: J Clin Med. 2021 Aug 16;10(16):3617. doi: 10.3390/jcm10163617 (PMC8396833; doi:10.3390/jcm10163617)
Supplement: Supplementary file 1 [file jcm-10-03617-s001.zip › jcm-1279913-supplementary.pdf]

Supplementary Table S1. Additional Cohort Characteristics.

Record ID (Sex)	Phenotype (*CRIM negative)	GAA Genotype		Age at Diagnosis (months)	Age at start of ERT (months)	Age at assessment (years)	ERT dose at assessment	Mobility status at assessment († with AFOs ** with walker)	Respiratory support at assessment	Primary nutrition/hydration source
		Allele 1	Allele 2							
1 (M)	IOPD	c.2481+102_2646+331del	p.Asn316Ser c.947A>G	0	0	6.2	40 mg/kg weekly	Nonambulatory	None	G-tube
2 (M)	IOPD	c.1438-1G>T (IVS09)	c.1655T>C (p.Lue552Pro)	0	2	17.0	40 mg/kg weekly	Nonambulatory	None	Oral
3 (M)	IOPD	c.1933G>A	c.1933G>A	2	3	15.3	30 mg/kg weekly	Ambulatory †	None	Oral
5 (M)	IOPD	C.1933G>A	c.1933G>A	1	2	12.1	40 mg/kg weekly	Nonambulatory	None	G-tube
6 (F)	IOPD	c.1802C>T p.Ser601Leu	c.1726G>A p.Gly576Ser	4	5	13.6	40 mg/kg weekly	Nonambulatory	BiPAP	G-tube
7 (F)	IOPD	c.655G>A)	c.655G>A)	6	6	11.1	40 mg/kg weekly	Nonambulatory	None	Oral
8 (F)	IOPD	c.1293_1312del20	c.1716C>G	1	1	7.8	40 mg/kg weekly	Ambulatory †	None	Oral
10 (M)	IOPD	c.2297A>C p.Tyr766Ser	c.2297A>C p.Tyr766Ser	7	7	13.8	40 mg/kg weekly	Nonambulatory	None	Oral
11 (M)	IOPD	c.1564	C>T p.P522S	6	6	6.6	40 mg/kg biweekly	Nonambulatory	BiPAP	G-tube
12 (M)	IOPD*	IVS2 +2_ +5 delTGGG deletion	c.1650_1651 dupG	7	7	9.8	40 mg/kg biweekly	Ambulatory	None	Oral
13 (F)	IOPD*	IVS2 +2_ +5 delTGGG deletion	c.1650_1651 dupG	0	0	7.3	20 mg/kg biweekly	Ambulatory	None	Oral
14 (F)	IOPD*	c.2560C>T p.Arg854X	c.2560C>T p.Arg854X	0	0	5.0	40 mg/kg weekly	Ambulatory	CPAP	Oral
16 (M)	IOPD	c.307T>G (p.C103G)	c.917C>T (p.S306L)	3	3	5.8	biweekly[u]	Ambulatory **	BiPAP	G-tube
17 (F)	IOPD	p.R702C	p.R702C	1	1	6.0	20 mg/kg weekly	Nonambulatory	BiPAP	Oral
18 (F)	IOPD	c.-32-13T>G	c.1841C>A	3	3	6.1	20 mg/kg weekly	Ambulatory	None	Oral

21 (F)	IOPD	c.925G>A (p.G309R)	c.1841C>A (p.T614K)	1	1	8.0	20 mg/kg biweekly	Nonambulatory	BiPAP	G-tube
27 (F)	IOPD	c.1447G>A	c.2560C>T	12	13	6.8	40 mg/kg weekly	Ambulatory †	None	Oral
15 (M)	LOPD	c.2501_2502delCA (p.T834fs)	c.-32-13T>G	26	27	9.3	20 mg/kg biweekly	Ambulatory	None	Oral
19 (M)	LOPD	c.-32-17_-32-10de-linsTCCCTGCTGAGCCTCC-TACAGGCCTCCCGC and	c.1447G>A	13	16	13.6	40 mg/kg weekly	Ambulatory †	None	Oral
20 (F)	LOPD	c.1978C>T	c.1477C>T; c.2221G>A	65	69	14.5	40 mg/kg weekly	Nonambulatory	BiPAP	Oral
22 (M)	LOPD	c.-32-13T>G	c.2560C>T	132	140	11.8	20 mg/kg biweekly	Ambulatory	None	Oral

IOPD=infantile onset Pompe disease; LOPD=late onset Pompe disease; CRIM=cross-reactive immunological status; ERT=enzyme replacement therapy; AFOs=ankle-foot orthotics; [u]= additional details unavailable. * CRIM negative; † with AFOs; ** with walker.

Supplementary Table S2. Physiologic Data from Sustained Phonation Tasks for Individual Study Participants.

Record ID (Sex/Age)	/s/ duration (s)	/z/ duration (s)	s/z ratio	MPT (s)
1 (M/6.2y)	0.93	2.44	0.38	2.02
2 (M/17.0y)	9.25	11.32	0.82	14.55
3 (M/15.3y)	4.87	5.27	0.92	8.65
5 (M/12.1y)	1.58	3.78	0.42	5.07
6 (F/13.6y)	1.06	0.93	1.13	0.89
7 (F/11.1y)	1.07	2.37	0.45	3.34
8 (F/7.8y)	1.53	1.42	1.08	2.01
10 (M/13.8y)	9.38	10.25	0.92	9.87
11 (M/6.6y)	0.89	1.65	0.54	3.76
12 (M/9.8y) +	1.76	2.59	0.68	7.4
13 (F/7.3y) +	4.79	4.91	0.98	10.77
14 (F/7.3y) +	0.94	1.19	0.79	1.48
16 (M/5.8y)	0.53	1.03	0.51	1.64
17 (F/6.0y)	0.63	0.69	0.92	0.67
18 (F/6.1y)	2.8	5.11	0.55	13.98
21 (F/8.0y)	1.64	1.86	0.88	3.02
27 (F/6.8y)	0.78	2.7	0.29	4.96
15 (M/9.3y) *	1.4	2.77	0.51	12.07
19 (M/13.6y) *	4.22	4.75	0.89	5.79
20 (F/14.5y *	1.95	0.96	2.03	6
22 (M/11.8y) *	8.31	9.48	0.88	-

+CRIM negative; *LOPD; MPT=maximum phonation time

Supplementary Table S3. Acoustic data from sustained phonation tasks for individual study participants.

Record ID (Sex/Age)	Jitter (%)	Shimmer (%)	Mean F0 (Hz)	Mean Loudness (dBA)	Irregularity	GNE Ratio	DSI
1 (M/6.2y)	0.05	10.68	319.84	93.08	0.85	0.38	-
2 (M/17.0y)	0.11	6.23	131.26	80.45	0.85	0.36	3.57
3 (M/15.3y)	0.11	7.82	129.65	81.03	0.93	0.21	0.1
5 (M/12.1y)	0.32	8.44	206.06	66.54	1.01	2.19	0.44
6 (F/13.6y)	0.37	5.39	291.6	70.87	1.06	0.87	-0.1
7 (F/11.1y)	0.1	5.37	217.17	85.87	0.81	0.29	0.24
8 (F/7.8y)	0.2	5.93	291.88	76.76	0.85	0.18	-2
10 (M/13.8y)	0.12	6.6	114.46	71.18	0.94	0.45	2.1
11 (M/6.6y)	1.59	21.34	306.11	80.12	1.8	0.54	-3.3
12 (M/9.8y) +	1.02	12.91	248.87	73.16	1.51	0.37	-
13 (F/7.3y) +	1.43	9.02	203.66	82.63	1.12	0.34	-3.1
14 (F/7.3y) +	0.69	8.97	283.38	81.15	1.09	0.42	-
16 (M/5.8y)	1.27	40.6	309.79	81.48	1.88	0.37	-2
17 (F/6.0y)	-	-	-	-	-	-	-
18 (F/6.1y)	2.59	27.47	254	73.03	2.01	0.48	-
21 (F/8.0y)	0.28	4.99	284.75	69.37	0.84	0.51	-1.6
27 (F/6.8y)	2.82	9.3	351.24	76.54	1.37	0.41	-5
15 (M/9.3y) *	0.16	9.82	225.05	73.65	0.97	0.27	-0.1
19 (M/13.6y) *	0.29	10.44	210.82	62.98	1.4	0.92	0.8
20 (F/14.5y *	0.13	8.06	202.85	67.71	0.96	0.22	0.3
22 (M/11.8y) *	0.07	4.88	309.56	89.05	0.73	0.49	-

[+] CRIM negative; * LOPD; F0=fundamental frequency; Hz=Hertz; dbA=decibels sound pressure level A-weighted (reference value = 20 μPa); GNE=glottal-to-noise-excitation; DSI=dysphonia severity index

Supplementary Table S4. Acoustic data from spoken text and maximal loudness tasks for individual study participants.

Record ID (Sex/Age)	Spoken Text (Rainbow Passage)			Maximum Loudness Task
	Mean Loudness (dBA)	Min Loudness (dBA)	Max Loudness (dBA)	Max Loudness (dBA)
1 (M/6.2y)	-	-	-	103.9
2 (M/17.0y)	69.79	61.8	76.6	94.3
3 (M/15.3y)	66.9	59.7	72.9	95.8
5 (M/12.1y)	64.36	59.6	66.9	80.6
6 (F/13.6y)	56.68	46	65.3	83
7 (F/11.1y)	73.52	61.4	81.19	95
8 (F/7.8y)	63.72	53.4	71.19	96.3
10 (M/13.8y)	64.57	51.8	72.9	97.6
11 (M/6.6y)	70.58	59.7	76.1	90.8
12 (M/9.8y)+	67.62	52.2	75.8	94.3
13 (F/7.3y) +	-	-	-	95.1
14 (F/7.3y) +	-	-	-	94.4
16 (M/5.8y)	-	-	-	87.7
17 (F/6.0y)	-	-	-	58.9
18 (F/6.1y)	-	-	-	87.2
21 (F/8.0y)	61.43	52.3	69	72.5
27 (F/6.8y)	-	-	-	101.3
15 (M/9.3y) *	66.27	59.2	71.3	104.3
19 (M/13.6y) *	59.22	47.5	65	89.6
20 (F/14.5y *	61.25	55.3	65.6	90.8
22 (M/11.8y) *	68.77	59.6	77	102.9

[+] CRIM negative; * LOPD; dbA=decibels sound pressure level A-weighted (reference value = 20 μPa)
